# When to Take a Break: Comparing Effects of Systematic Short (Pomodoro) and Self-Regulated Breaks on Subjective Experience and Actual Learning

**DOI:** 10.3390/bs16071158

**Published:** 2026-07-09

**Authors:** Ali Göksu, Wisnu Wiradhany, Anique B. H. de Bruin

**Affiliations:** 1 Department of Educational Research & Development, School of Health Professions Education, Faculty of Health, Medicine, and Life Sciences, Maastricht University, 6211 LK Maastricht, The Netherlands; anique.debruin@maastrichtuniversity.nl; 2 Faculty of Psychology, Atma Jaya Catholic University of Indonesia, Jakarta 12930, Indonesia; wisnu.wiradhany@atmajaya.ac.id

**Keywords:** break-taking, systematic short breaks, moods, learning performance

## Abstract

Despite the potential of break-taking to regulate study effort, mitigate fatigue, and increase positive affect during learning, few studies have experimentally investigated its effects on students’ learning experiences and performance. This study investigates the effects of two break strategies, namely systematic short breaks (externally structured and predetermined, e.g., the “Pomodoro” technique) and self-regulated breaks (self-decision on when to take a break) on university students’ fatigue, moods, learning outcomes and overall experience. One hundred seventy-six university students completed a reading task on climate change, during which they repeatedly rated their mood, took a comprehension test, and answered open-ended questions under one of two break conditions. The results revealed no significant difference in performance between the two break strategies, although the sensitivity of the performance measure may have been limited. Self-regulated breaks led to more positive moods, including higher energy, concentration, and motivation to study, and lower distraction. The study suggests that flexibility in break-taking may be important for tailoring break strategies to students’ needs and specific learning tasks, while also indicating that the effectiveness of systematic breaks may depend on the specific timing and duration of the break schedule.

## 1. Introduction

Students in higher education often face challenges such as prolonged study periods and the mental fatigue that accompanies intensive learning tasks. Fatigue, which arises from sustained engagement in cognitively demanding activities, can significantly hinder students’ learning and performance. To maintain effective learning, students should manage their cognitive resources efficiently by employing strategies that prevent or mitigate mental fatigue (Biwer et al., 2023; Gilsoul et al., 2022).

In the context of mental fatigue mitigation, effort regulation emerges as a crucial skill for enhancing students’ learning outcomes (Biwer et al., 2023). As a key component of Self-Regulated Learning (SRL), effort regulation involves strategic decision-making about when to initiate, sustain, or adjust effort in learning tasks (de Bruin et al., 2020). SRL provides a comprehensive framework for understanding these processes, emphasizing how learners actively plan, monitor, and regulate their cognitive, behavioral, and motivational resources to achieve their goals (Zimmerman, 2002). Within this framework, effort regulation involves not only maintaining performance and managing cognitive resources but also making strategic decisions about when to pause and recover from mental fatigue (Hockey, 2013). Break-taking has therefore emerged as an effective strategy for regulating effort through which learners temporarily pause study activity to recover cognitive and motivational resources (Biwer et al., 2023; de Bruin et al., 2020). However, although there have been advances in understanding how and why students decide to invest or not invest effort in their learning, little is known about why students choose to pause their effort investment and take a break during their learning. This is surprising because breaks have been shown to reduce the detrimental effects on performance caused by prolonged task performance (Helton & Russell, 2017).

Break-taking is commonly categorized into two forms: systematic breaks, which involve externally structured intervals (e.g., “Pomodoro” technique; Cirillo, 2018), and self-regulated breaks, which consist of unstructured pauses initiated by learners’ subjective judgments during learning (Biwer et al., 2023). Viewed through the lens of SRL, systematic breaks provide external structure that may support effort regulation by reducing the need for students to continually monitor and decide when to pause during learning. In digital learning contexts, growing evidence suggests that integrating systematic breaks into technology-enhanced environments such as video-based or online learning may enhance their effectiveness by supporting cognitive load management, fatigue reduction, and sustained engagement (Albulescu et al., 2022; Biwer et al., 2023; Ogut, 2025). Despite growing interest in break-taking, findings on systematic and self-regulated breaks remain mixed, which may be due to differences in task characteristics, outcome measures, and break schedules. Importantly, most experimental studies have examined students’ own self-selected study tasks in authentic learning environments and therefore could not objectively measure learning outcome effects (Biwer et al., 2023; Smits et al., 2025). This makes it difficult to determine whether differences between break conditions are due to the break format itself or to variation in task type, task difficulty, and study goals. Unlike studies in which students worked on self-selected tasks, all participants in the present study completed the same reading task under the same online study conditions. This study examined whether differences between break strategies emerge when task content, study materials, and outcome measures are held constant. By assessing both objective learning performance and subjective learning experiences, including mood, fatigue, and students’ perceptions of break-taking during a common learning task, the present study aimed to determine whether systematic and self-regulated breaks differ in their effects on learning outcomes and actual learning experiences during self-study.

### 1.1. Effort Regulation and Recovery

Self-Regulated Learning (SRL) is a dynamic process through which learners actively regulate their cognitive, behavioral and motivational strategies to achieve their learning goals (Zimmerman, 2002). It encompasses various components, including goal setting, strategic planning, monitoring, and adjusting learning behaviors. Within this framework, effort regulation emerges as a critical component that enables learners to navigate the challenges of sustaining attention and motivation during demanding tasks. Effort regulation also involves learners’ decisions to initiate, sustain, or stop effort in learning tasks (de Bruin et al., 2020). This process is not static; instead, it requires learners to continuously evaluate their cognitive and motivational resources and adapt their strategies to sustain performance (Grund et al., 2024; Lohbeck & Moschner, 2022). Such a dynamic process is also crucial in self-study environments, where strategies to manage mental effort effectively play a key role in maintaining performance (Gorbunova et al., 2024). However, sustaining effort over extended periods is especially challenging during cognitively demanding tasks, such as reading comprehension, which deplete working memory resources (Biwer et al., 2023). Here, prolonged mental effort may result in directed attention fatigue, which impairs performance and learning (Kaplan, 1995).

Effort-Recovery Theory (Meijman & Mulder, 1998), which emphasizes the necessity of timely pauses to restore depleted resources, suggests that sustained effort during work results in load reactions such as fatigue, cognitive overload, or physiological activation which can be recovered during periods of rest. According to this theory, to recover effectively, individuals need to mentally disconnect from work during their time off. This psychological detachment is crucial for recovery, as it allows the mind and body to rest and prevents prolonged fatigue (Sonnentag & Fritz, 2007). As a result, individuals can replenish internal resources such as energy, positive mood, and motivation, which are essential for restoring balance and protecting against further resource depletion (Baktash & Pütz, 2025; Sonnentag & Fritz, 2007).

Building on this recovery perspective, an effort-regulation framework conceptualizes break-taking as a recovery strategy through which learners regulate their cognitive effort. From this viewpoint, breaks are not merely passive recovery periods but deliberate actions that enable learners to disengage from task demands, restore resources, and sustain performance over time (Sonnentag & Fritz, 2007; Zijlstra & Sonnentag, 2006; Zhou et al., 2026). In light of these benefits, incorporating break-taking supports sustained effort regulation across extended periods of learning. Accordingly, by externally interrupting continuous task engagement, breaks allow learners to mitigate fatigue and replenish energy, enhancing their ability to sustain focus and productivity (Katidioti et al., 2016; Sonnentag & Fritz, 2007). Building on these theoretical perspectives, break strategies appear as a critical tool for managing effort regulation effectively, mitigating fatigue, and replenishing energy.

### 1.2. Break-Taking in Learning

Break-taking is widely recognized as an effective strategy for mitigating the adverse effects of prolonged mental effort and supporting the regulation of cognitive resources (Biwer et al., 2023). Ample research demonstrates that incorporating breaks during task performance helps replenish cognitive and emotional resources, maintain motivation, and reduce fatigue, thereby enabling individuals to sustain prolonged engagement and maintain performance during cognitively demanding activities. (Albulescu et al., 2022; Kim et al., 2017, 2022; Zhu et al., 2019).

From a cognitive load perspective, break-taking serves as a recovery strategy that enables learners to manage cognitive demands by disengaging from tasks, replenishing cognitive resources, increasing concentration and mitigating fatigue to sustain performance (Albulescu et al., 2022; Biwer et al., 2023; Sharpe et al., 2025; Zhou et al., 2026). Consistent with this view, recent research demonstrated that breaks could temporarily reduce cognitive load during periods of high task demand, enabling learners to regulate mental effort more effectively, facilitate resource recovery, and ultimately improve task performance (Lee et al., 2020; Sharpe et al., 2025). A recent meta-analysis also indicated that breaks preserve vigor and alleviate fatigue, thereby enhancing overall well-being and performance outcomes in cognitive task demands (Albulescu et al., 2022). Taken together, these findings suggest that break-taking can be not only a passive pause in study sessions but also an active strategy for effort regulation. By taking breaks, learners can step away from task demands, restore both cognitive and emotional resources, manage their effort more effectively, and return with renewed energy and focus. As a result, this process supports sustained effort over time and enhances performance by mitigating mental fatigue (Adler & Benbunan-Fich, 2013).

Beyond its impact on task performance, break-taking also plays a crucial role in regulating moods. Mood is defined as “a set of feelings, ephemeral in nature, varying in intensity and duration, and usually involving more than one emotion” (Lane & Terry, 2000, p. 7). Research suggests that break-taking serves as a key strategy for mood regulation by reducing negative affect such as distraction and fostering positive states such as energy (Biwer et al., 2023). Grounded in Effort–Recovery theory (Meijman & Mulder, 1998), breaks function as a recovery mechanism that enables individuals to regain internal resources, including energy, positive mood, and motivation. By facilitating emotional recovery, breaks support mood regulation while also restoring cognitive and motivational resources and increasing the concentration necessary for effective task engagement and effort regulation (Owen & Watts, 2026; Sonnentag & Fritz, 2007; Zhu et al., 2019; Zhou et al., 2026). Empirical evidence shows that break-taking helps individuals recover from negative mood affect such as fatigue and boredom, enhances positive mood and energy, and sustains motivation by relieving cognitive strain (Adler & Benbunan-Fich, 2013; Biwer et al., 2023; Sonnentag & Zijlstra, 2006).

Research also indicates that break-taking in online learning environments improves concentration, reduces fatigue, and supports more sustainable engagement over time (Albulescu et al., 2022; Biwer et al., 2023; Ogut, 2025). For example, Albulescu et al. (2022) found that incorporating breaks into digital learning increased learners’ vigor, alleviated fatigue, and improved focus. Their findings further suggest that by enhancing emotional well-being and reducing fatigue, breaks not only improve learners’ immediate learning experience but also contribute to longer-term psychological resource gains. Together, these perspectives provide a theoretical basis for understanding how breaks function as recovery strategies that support both affective regulation and sustained performance; however, the use of varying break strategies across existing studies makes it difficult to identify the boundary conditions under which breaks are most effective for a common task.

### 1.3. Systematic and Self-Regulated Break Strategies

Breaks can be systematic (e.g., “Pomodoro” technique; Cirillo, 2018) involving externally structured and predetermined intervals for study and rest, or self-regulated, consisting of unstructured or unplanned pauses initiated by learners’ subjective assessments and occurring irregularly and unpredictably during learning (Biwer et al., 2023; Smits et al., 2025). The systematic breaks incorporate both short and long structured breaks, with short breaks providing brief pauses between study cycles and long breaks allowing extended recovery (Biwer et al., 2023). Research suggests that systematic breaks promote effective effort regulation and facilitate recovery by reducing cognitive load and fatigue, and removing the need for continuous self-monitoring and decision-making about break timing (Biwer et al., 2023; Katidioti et al., 2016; Lee et al., 2021; Ogut, 2025). More recently, Owen and Watts (2026) showed that structured and systematic breaks lead to greater engagement, a better connection with the material, and make it easier for students to remain focused. In contrast, self-regulated breaks offer greater flexibility and autonomy, potentially enhancing learners’ sense of agency and personal control. However, because self-regulated breaks tend to occur irregularly and unpredictably, they may often be initiated too late to provide optimal restorative benefits (Bosch & Sonnentag, 2018).

Recent empirical studies, however, suggest a more nuanced picture regarding the benefits of systematic versus self-regulated break approaches. For example, Ogut (2025), in a recent review, found that systematic (Pomodoro) breaks improve learners’ focus, reduce mental fatigue, enhance engagement, and promote better time management compared to self-regulated study and break. In contrast, Smits et al. (2025), in their comparison of self-regulated, Pomodoro, and flowtime break-taking techniques, found that structured (Pomodoro) breaks were associated with a faster increase in fatigue and a faster decline in motivation over time compared with self-regulated breaks, although no significant differences emerged in productivity, task completion, or flow across conditions. In another recent study, Biwer et al. (2023) examined how three study-break strategies—self-regulated breaks, systematic long breaks (24 min study + 6 min break), and systematic short breaks (12 min study + 3 min break)—affected university students’ mental effort, mood, and task performance during online self-study environments. While both systematic conditions led to lower mental fatigue, reduced distraction, and improved mood compared to self-regulated breaks, no significant differences were observed in mental effort or task completion across the groups. Critically, it remains unclear to what extent different break benefits also contribute to task performance, as students chose their own learning goals/tasks and their learning outcomes were not measured. To truly estimate the potential of systematic breaks it is crucial to examine whether systematic breaks have a positive effect on learning outcomes. Therefore, research is needed that controls the learning task and the assessment. This highlights a critical gap in understanding how standardized measures of learning outcomes and task consistency influence the benefits of different break types (systematic vs. self-regulated), learners’ break experiences, and overall learning performance.

### 1.4. The Present Study

Previous research has indicated that students engaging in systematic short and long breaks reported lower levels of mental fatigue and distraction compared to those taking self-regulated breaks (Biwer et al., 2023). However, significant questions remain regarding how these break strategies influence task performance and learner experiences, particularly in contexts where task selection is externally controlled (cf. Biwer et al., 2023). To address this, we standardized the learning task (reading a text) across all conditions, which ensured consistent task demands and task characteristics across conditions to compare performance outcomes across break conditions. In the present experiment, we aimed to simulate a more natural study-and-break pattern informed by recent empirical findings on task switching and break-taking during online activities. Previous research suggests that individuals tend to switch between tasks approximately every 12 min during online activities as attention declines after this interval, while a 3 min break represents half the duration of the standard 25–5 min Pomodoro break structure commonly used in the literature (Biwer et al., 2023; Buser & Peter, 2012; Gonzalez & Mark, 2004; Gould et al., 2013; Mark et al., 2005). These findings provide a rationale for the systematic short-break design used in the present study. Therefore, we used a 12–3 learning-break pattern in the systematic short-break condition, consisting of 12 min of online studying followed by a 3 min break. In addition, despite emerging evidence on study-and-break patterns, few studies have systematically examined the effects of systematic short breaks versus self-regulated breaks on students’ learning performance and overall study experiences. Moreover, it is still unclear how students’ mental fatigue and moods may vary as a function of task characteristics, especially when they engage in predetermined learning tasks rather than self-selected tasks. To address these gaps, this study compared systematic short and self-regulated breaks in terms of their effects on university students’ learning outcomes, mood, mental fatigue, and overall study experiences. We formulated the following research questions and hypotheses:

- Research Question 1: What is the effect of systematic short versus self-regulated break-taking on students’ learning outcomes and mood states?

**Hypothesis** **1a.** *Students who take systematic short breaks would perform better in their learning outcomes and score higher on the retention test compared to students who take self-regulated breaks.*

**Hypothesis** **1b.** *Students who take systematic short breaks would experience less fatigue, reduce negative affect, and increase positive affect compared to students who take self-regulated breaks.*

- Research Question 2: How do students experience and evaluate (systematic short vs. self-regulated) break interventions during their self-study?

## 2. Methods

This study was approved by the Ethics Committee of the Faculty of Health, Medicine and Life Sciences (FHML) at Maastricht University (Identification code: FHML-REC/2023/127; date of approval: 9 January 2024). We also preregistered the study’s hypotheses, planned methods, and planned analyses to enhance transparency and reproducibility. The preregistration is available on the Open Science Framework at https://doi.org/10.17605/OSF.IO/UBNXS (accessed on 6 July 2026).

### 2.1. Participants

To estimate the required sample size, we conducted an a priori power analysis in G*Power 3.1 (Faul et al., 2009) for an independent-samples *t*-test with an alpha level (α) of 0.05 and a statistical power of 0.80. An effect size of *d* = 0.4 (Cohen, 1988) was used, following recommendations that this value reflects a typical effect size in behavioral and psychological research (Brysbaert, 2019). The results showed that a total of 156 participants (N = 78 per condition) would be sufficient to detect the effectiveness of the two break conditions. Four hundred and sixty university students registered for participation and gave their informed consent online.

Of 460 participants who registered and provided informed consent, 205 participants completed the study. Following our preregistered exclusion criteria, 29 participants were excluded from the main analyses. Specifically, 18 participants assigned to the systematic-break condition were excluded because they failed to comply with the study instructions, including adhering to the prescribed study duration and taking at least one break within the preregistered criteria. In addition, 9 participants were excluded due to excessively short reading times (more than 3 *SD* below the mean reading duration, calculated using the Flesch–Kincaid readability formula; Kincaid et al., 1975), and 2 participants were excluded for completing the reading comprehension test in an unusually short time. After these exclusions, the final sample comprised 176 participants for analysis. Participants included 122 bachelor’s and 54 master’s degree students from different study programs/faculties (Health Science, Biomedical Science, Economics and Business, Law and Psychology) at Maastricht university. The participants, aged between 18 and 41 years (*M* = 21.62, *SD* = 2.74), consisted of 134 females (76.1%), 41 males (23.3%), and 1 participant (0.6%) who indicated “other.” They were randomly assigned to one of the break conditions that was programmed in Qualtrics: systematic short breaks (N = 90) and self-regulated breaks (N = 86). All participants received €20 in exchange for their participation.

Study participation and compliance were monitored using time-stamped process data automatically recorded by the Qualtrics platform for all study and break sessions. Compliance with the assigned protocols was assessed based on study time, break duration, break frequency, and completion of study blocks. In the systematic break condition, participants were automatically redirected to a break page after each study block. However, they were allowed to proceed to the comprehension test once they had finished reading the text, taking into account that some participants were able to read the text quickly and complete the reading task before reaching the fourth study block. Therefore, minor variation in break-taking reflected differences in reading speed, the number of break-takings and completed study blocks (i.e., total task engagement), rather than missed timers or protocol deviations. Participants who failed to take at least one scheduled break or otherwise showed clear non-compliance with the study procedure were excluded from the main analyses in accordance with the preregistered criteria. In the self-regulated condition, variability in break behavior was expected by design, as participants could freely decide when and how long to take breaks. Compliance indicators, including study time, break duration and frequency, were also reported in the Section 3.

### 2.2. Materials

#### 2.2.1. Reading Task

All participants in both conditions studied a common text about ‘Ground water and climate change’ (Taylor et al., 2013) which contained 4599 words (Flesch-Kincaid grade level of 16.7). The Flesch-Kincaid readability formula (Kincaid et al., 1975) estimated an average reading time of 23 min for this text.

#### 2.2.2. Reading Comprehension Test

To assess learning outcomes, participants completed a 20-item multiple-choice reading comprehension test consisting of 10 factual and 10 conceptual items, each with four response options. The items were generated using an OpenAI-based approach (OpenAI, 2024) based on the content of the target reading text (Taylor et al., 2013). ChatGPT-4 was prompted to generate multiple-choice questions that assessed both factual recall and conceptual understanding. The factual items assessed recall of specific information from the text, whereas the conceptual items assessed students’ understanding of broader ideas and relationships presented in the text. The generated items were then reviewed, revised, and refined by the first author to ensure alignment with the reading text, conceptual accuracy, appropriate difficulty, and clarity of wording. The correctness of the response options and answer keys was also manually checked against the source text. Sample reading comprehension test items are provided in Appendix A.

The study material and test items were piloted with 15 bachelor’s and master’s students from various study programs at the same university. Based on their feedback, minor revisions were made to improve the clarity, wording, and accuracy of the materials and assessment items. Basic item statistics indicated sufficient variability in item difficulty that ranges from M = 0.38, *SD* = 0.49 (item 18, the most difficult) to M = 0.85, *SD* = 0.36 (item 2, the easiest), demonstrating adequate sensitivity to performance differences. The reliability of the test scores was measured, yielding a Cronbach’s alpha of 0.54. Even though this value indicates low internal consistency, we did not remove additional items solely to increase the alpha coefficient. Cronbach’s alpha is influenced by the dimensionality of the instrument and may not accurately reflect reliability when its underlying assumptions are violated (Sijtsma, 2009). When a test assesses multiple content domains or skills, alpha may not accurately reflect reliability and may underestimate reliability for multidimensional measures (Widhiarso & Ravand, 2014). In the present study, the reading comprehension test was designed to assess multiple aspects of text understanding rather than a single narrow skill, which may help explain the relatively low alpha coefficient.

#### 2.2.3. Mood Questionnaire

Students’ moods were assessed using a brief self-report questionnaire adapted from prior research (Biwer et al., 2023; Milyavskaya et al., 2019). Participants were asked, “How are you feeling right now?” and rated their current state across eight mood dimensions, comprising four positive mood-related dimensions (energy, concentration, interest, and motivation) and four negative mood-related dimensions (fatigue, distraction, boredom, and stress) (D. Watson et al., 1988). After each break, participants repeatedly rated each mood dimension using a 5-point Likert scale ranging from 1 (not at all) to 5 (very much). Each dimension was assessed using a single-item indicator (e.g., “fatigued,” “energized,” “distracted”). Reliability analyses across repeated measurements indicated excellent internal consistency for both positive and negative mood-related dimensions, with Cronbach’s alpha coefficients of 0.91 and 0.88, respectively.

#### 2.2.4. Evaluation of Break Intervention

The intervention questionnaire, adapted from Weiner et al. (2017) and Keating et al. (2022), assessed the acceptability and feasibility of break-taking. At the end of all study sessions, participants rated their agreement on a 5-point Likert scale (1 = Strongly Disagree, 5 = Strongly Agree). The scale captured perceptions of the intervention using the following statements: “This way of break-taking is acceptable to me,” “This way of break-taking is appealing/attractive,” “I like this way of taking breaks,” “This way of break-taking seems implementable,” and “This way of break-taking seems easy to use.” The scale demonstrated strong internal consistency, with a Cronbach’s alpha of 0.84.

Additionally, participants were asked to reflect on their subjective experiences and perceptions of systematic short breaks versus self-regulated breaks in relation to their overall study process through the following six open-ended questions: “How was the current break-taking in this online study different from your usual studying?”, “How did this break-taking influence your study?”, “Have you experienced any advantage(s) of current break-taking in your online self-study?”, “Have you experienced any disadvantage(s) of current break-taking in your online self-study?”, “Is this break-taking applicable for all your studies?”, and “Do you have any other comments or suggestions regarding the intervention?”.

### 2.3. Procedure

**Figure 1:**
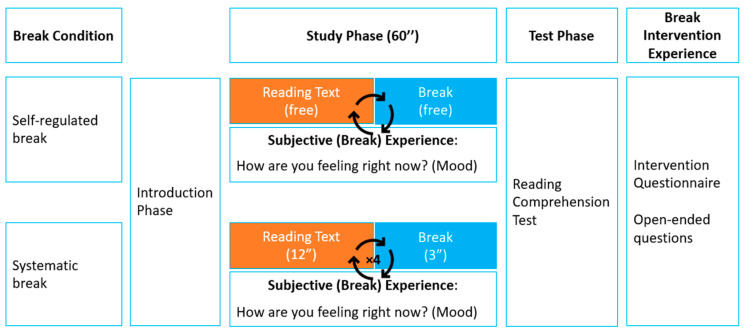
An overview of the study procedure.

Data was collected through a Qualtrics online environment, where participants engaged in the study remotely using either a computer or laptop. Figure 1 shows an overview of the study procedure. Upon providing informed consent, participants filled out their demographic details such as age, gender and study program. Afterwards, they were randomly assigned to one of two break conditions (systematic short or self-regulated). In the instruction phase, all participants received general instructions on navigating all phases of the study, including guidance on taking breaks, resuming the study, responding to questions, and completing the questionnaires. Participants in each condition received a somewhat different set of instructions that corresponded to the break strategies they follow. Additionally, participants were reminded of the overall time limit for completing the study and encouraged to approach the tasks and breaks in a realistic manner.

Participants in the systematic-break condition were instructed to study the text for up to four study blocks within a total of 60 min. Each block consisted of 12 min of reading time followed by a 3 min break. These study and break lengths were based on previous research on interruption patterns and task switching (Biwer et al., 2023; Buser & Peter, 2012; Gonzalez & Mark, 2004; Mark et al., 2005; Gould et al., 2013). Participants who finished reading the text before completing all four study blocks were allowed to proceed directly to the reading comprehension test. Otherwise, after every 12 min of study, the study page automatically changed to a break page, where a timer displayed the remaining time of the 3 min break. During breaks, participants could do anything they wanted. Following each break, participants repeatedly completed a mood questionnaire assessing their current mood states and a short follow-up question about their break activity (“What did you do during your break?”) (Fritz et al., 2011). After completing the questionnaire, the participants continued studying the same text in the following study block.

In the self-regulated break condition, participants studied the same text for as long as they wished. They freely initiated their break at any time during reading by clicking the ‘take a break’ button and then engaged in any activity during their break period. Each study block in this condition consisted of a period of continuous reading immediately followed by a self-initiated break. At the end of each break, they clicked the ‘continue to study’ button and, like the other group, repeatedly completed the same mood questionnaire as well as a short follow-up question about their break activity (Fritz et al., 2011). After completing both questionnaires, participants resumed studying the same text.

After completing all study blocks, participants in both conditions answered a reading comprehension test including 20 multiple-choice questions based on the text. In the final phase of the study, the participants completed an intervention questionnaire assessing the acceptability and feasibility of their breaks. They also responded to six open-ended questions about their study experiences with the breaks.

### 2.4. Data Analysis

All statistical analyses were conducted using SPSS 31. Independent sample t-tests were conducted to evaluate differences in mood, learning outcomes, and the acceptability and feasibility of the two break strategies based on intervention questionnaire data. For the mood analyses, repeated ratings for each mood dimension were averaged across measurement occasions for each participant. Due to our unbalanced design (i.e., the number of breaks participants may take in the self-regulated condition varies) and low level of variability in the number of breaks participants took in the self-regulated condition (i.e., most participants only took one break), we did not analyze the data using a repeated-measures or multilevel analysis approach, respectively, to avoid Type I and II error inflation (e.g., see Oberpriller et al., 2022). Moreover, one-tailed tests were used for all planned comparisons, and *p*-values were adjusted using the false discovery rate (FDR) procedure to control for multiple comparisons (Benjamini & Hochberg, 1995). The qualitative data collected from the open-ended questions was analyzed using Atlas.ti 25, which provided thematic analysis by coding responses (Braun & Clarke, 2006). Accordingly, participants’ responses were analyzed separately for each condition and across conditions to identify both overarching and condition-specific themes by using a coding procedure. To enhance transparency and coding reliability, independent coding, blind review, and iterative consensus discussions were employed throughout the coding process. Coding decisions were independently and continuously reviewed and refined by multiple researchers. Students’ responses were divided into three datasets (33%, 33%, and 34%, respectively). The first author and an external blind reviewer, not part of the research team, independently coded the first dataset and refined the coding framework. They then collaboratively reviewed all codes in the first dataset and compared their coding within each break condition. Then the first author applied these codes to the second dataset, and the blind reviewer reviewed these codes in the second dataset to ensure consistency. Percentage agreement across the codes for each open-ended question was used as an inter-coder agreement statistic to evaluate the consistency between the first author and the blind reviewer. Discrepancies were discussed and resolved through consensus, resulting in minor refinements to the coding framework. The finalized codes were then applied by the first author to the remaining responses across both conditions, identifying differences between groups. Finally, the first and second authors jointly reviewed all coding in both groups to ensure overall consistency and accuracy. After completing the coding process, ATLAS.ti 25 was used to compare the codes for each open-ended question across two break groups. This thematic analysis provided deeper insights into how students perceived the systematic short versus self-regulated breaks and evaluated their experiences during the self-study sessions and how these break strategies influenced their overall learning and mood states. Common themes and notable remarks were reported, with participant responses anonymized using numerical identifiers (e.g., P1, P2, P3) within each break condition to ensure confidentiality. To enhance methodological transparency, the final coding framework, including the main themes, related codes, and illustrative quotes, is also presented in Appendix B.

## 3. Results

### 3.1. Break Activities

Participants in both groups engaged in a variety of activities during breaks. The most common activity was checking social media or sending personal messages on social media (49.64%), followed by drinking water, coffee, or tea (22%). Additionally, some participants reported engaging in ‘other’ break activities, including some physical activities, just taking a rest, or chatting with friends (18.52%), while others used the break to go to the WC/bathroom (9.84%).

### 3.2. Quantitative Results: Effects of Break-Taking on Students’ Mood and Reading Comprehension Test

Table 1 shows that the average duration of individual study sessions (learning time) did not significantly differ between systematic and self-regulated groups even though participants engaged in different study–break formats, *t*(174) = −0.23, *p* = 0.408, *d* = 0.035, 95% *CI* [−0.33, 0.26]. As expected, participants following fixed breaks in the systematic break condition took significantly more breaks overall than those in the self-regulated condition, *t*(174) = 8.52, *p* = 0.001, *d* = 1.28, 95% *CI* [0.96, 1.61]. Participants in this systematic condition were allowed to proceed to the comprehension test once they had finished reading the text, as some participants were able to read the text quickly and complete the reading task before reaching the fourth study block. Thus, minor variation in the scheduled break frequency reflected differences in reading speed, completed study blocks, and overall task engagement rather than missed timers or protocol deviations. Despite the differences in break frequency across two break conditions, average break length per participant (in minutes) did not differ significantly between the two conditions, *t*(174) = −1.54, *p* = 0.063, *d* = 0.23, 95% *CI* [−0.53, 0.065]. Break-lengths in the systematic condition were fixed by design, whereas break durations in the self-regulated condition showed more variability, reflecting participants’ flexibility in break timing.

In addition, contrary to our preregistered hypothesis 1a, *t*-test results (Table 1) did not reveal a statistically significant difference in reading comprehension test scores between the two break conditions, *t*(174) = 1.60, *p* = 0.056, *d* = 0.24, 95% *CI* [−0.055, 0.54]. To further assess evidence for the absence of a meaningful difference, we conducted a Bayesian independent-samples *t*-test using the Rouder method. The analysis yielded a Bayes factor of *BF*_01_ = 2.49, suggesting the observed data were approximately 2.5 times more likely under the null hypothesis than the alternative. This provided anecdotal to moderate evidence supporting the absence of a difference in reading comprehension performance between the groups.

In contrast to our preregistered Hypothesis 1b, systematic short breaks also did not provide a notable advantage over self-regulated breaks in improving mood states during self-study sessions. To control for Type I error across multiple mood comparisons, *p*-values were corrected using the FDR procedure (Benjamini & Hochberg, 1995). The *t*-test results (Table 2) revealed advantages of self-regulated breaks in certain mood states, with participants reporting higher energized, *t*(174) = −2.37, *p* = 0.020, *d* = −0.36, 95% *CI* [−0.65, −0.06]; more concentrated, *t*(174) = −2.86, *p* = 0.005, *d* = −0.43, 95% *CI* [−0.73, −0.13]; and more motivated to study, *t*(174) = −3.56, *p* = 0.002, *d* = −0.54, 95% *CI* [−0.84, −0.24], while also experiencing less distraction, *t*(174) = 3.63, *p* = 0.004, *d* = 0.55, 95% *CI* [0.25, 0.85], compared to those in the systematic short condition. However, no significant differences were observed for participants’ moods of interest (*d* = −0.19), fatigue (*d* = 0.050), boredom (*d* = 0.22), and stress (*d* = −0.055) (all *p*s ≥ 0.05, 95% *CI*s included zero).

To address RQ2, we first conducted quantitative analyses of students’ self-reported evaluations of the break interventions, comparing systematic short and self-regulated breaks across multiple acceptability and feasibility dimensions. *p*-values were also adjusted using the FDR procedure (Benjamini & Hochberg, 1995). As shown in Table 3, self-regulated breaks gained higher acceptance, *t*(174) = −2.78, *p* = 0.005, *d* = −0.42, 95% *CI* [−0.72, −0.12]; attractiveness, *t*(174) = −3.73, *p* = 0.003, *d* = −0.56, 95% *CI* [−0.86, −0.26]; and preference, *t*(174) = −4.23, *p* = 0.003, *d* = −0.64, 95% *CI* [−0.94, −0.33], whereas no significant difference was found for implementability (*d* = −0.26) and usability (*d* = −0.21), (*p* ≥ 0.050, 95% *CI*s included zero).

### 3.3. Qualitative Results: Students’ Experiences on Break-Taking

As part of addressing RQ2, the qualitative analysis identified several significant themes regarding participants’ experiences with self-regulated and systematic short breaks. These themes included: (1) differences and similarities in participants’ break-taking behaviors, (2) the influence of breaks on their study efficiency and focus, (3) perceived advantages and (4) disadvantages of taking breaks, and (5) the practical application of break strategies within participants’ self-study routines.

#### 3.3.1. Differences and Similarities in Break-Taking

A notable finding was that participants in both conditions preferred longer, less frequent breaks, including those in the systematic short break condition, for whom this preference contrasted with the shorter, more frequent break structure imposed by the study. For example, in the systematic short break condition, instead of taking frequent three-minute breaks, several participants strongly preferred longer breaks, feeling that the imposed short, frequent breaks disrupted their focus and productivity, noting; ‘*Usually I tend to study for a longer duration and then also take a longer break; for example, study around 45 min, then take a 10–15 min break*’ (P22). In the self-regulated condition, participants also generally exhibited a preference for longer, less frequent breaks, aligning closely with their habitual study practices and personal time-management strategies. Most students in this condition expressed a preference for maintaining their established break routines, with comments such as, ‘*I do not usually take many breaks while studying, because I want to get the task done most efficiently*’ (P28) and ‘*I usually take breaks less frequently*’ (P13).

#### 3.3.2. Break Influences on Study Efficiency and Focus

The analyses showed that break-taking emerged as a significant factor influencing participants’ study efficiency and focus, with notable differences between the two conditions. Several participants taking systematic short breaks often expressed that the frequent breaks had a negative impact on their study flow, disrupting their concentration, remarking, ‘*I found it [systematic short break] too short, my concentrations were broken each time*’ (P08). However, a few participants found that the systematic short breaks provided short moments of renewed focus: ‘*I think I was more focused because I could take mini breaks*’ (P73). On the other hand, many participants in the self-regulated group highlighted how breaks allowed them to manage their energy levels effectively, facilitating sustained concentration on their tasks over extended periods, noting: ‘*It [self-regulated break] allowed me to regain concentration to read again. The text was quite long, so I needed the break to stay focused*’ (P81).

#### 3.3.3. Perceived Advantages of Break-Taking

Regarding the advantages of the breaks, some participants found that systematic short breaks helped them maintain motivation, focus and concentration over time, expressing (P05); ‘*taking [systematic short] breaks more often did help me focus more, because I told myself I ‘only’ had to focus for 12 min before I could do something fun*’. However, some participants reported that the frequent breaks in the systematic short breaks had little or no advantage on their learning productivity, remarking; ‘*Personally, this method [systematic short breaks] of taking breaks has no advantage for me. My usual break-taking is far more fruitful as it allows me to ‘cleanse’ my mind from the previous topic and prepare it for the following one…*’ (P35). On the other hand, in the self-regulated break group, a great number of participants expressed that those self-regulated breaks enhanced their motivation and productivity, noting: ‘*Yes, it [self-regulated break] gives me more energy to continue with studying*’ (P61), and *‘[self-regulated break] increased long-span concentration, increased long-span motivation, [provided] less boredom*’ (P34). However, some participants also emphasized that the self-regulated breaks did not significantly give a visible advantage on their performance.

#### 3.3.4. Perceived Disadvantages of Break-Taking

Despite reporting some advantages, many participants in the systematic short break condition noted specific challenges related to the timing of the breaks, rather than break-taking itself. They highlighted that the imposed short and frequent breaks disrupted their study flow, not because breaks were ineffective, but because the duration was too limited to engage in meaningful rest activities, such as making tea or using the restroom. As one participant (P35) taking systematic short breaks observed, ‘…*the time to study is very short, so when one actually starts understanding what is going on in the reading material, the time is usually up and the break deflates all the interest/motivation to go on. The break itself is too short to do any significantly different activities…*’. On the other hand, over half of the participants in the self-regulated condition reported no disadvantages to their self-regulated breaks, remarking; *‘I have not [had any disadvantages], this was very effective for me’* (P46). However, some participants also noted that self-regulated breaks negatively affected their productivity and increased distractions, stating; *‘[during break], I feel that I waste my time too much’* (P23), and *‘[break] breaks up my thought processes and invites more distractions*’ (P76).

#### 3.3.5. Break Applicability to Study Routine

Participants in both conditions offered varied perspectives on the applicability of self-versus systematic short breaks to their study routines. On one hand, half of the participants emphasized the conditional usefulness of systematic short breaks, noting that their effectiveness varied with the context; ‘*I think it depends. For readings articles and books, it [systematic short break] is a nice way to recharge as you can get tired from reading for long sessions but for other assignments such as writing, it is nice to just let the ‘study time’ flow on until I feel that I need a break, rather than having pre-planned breaks*’ (P22). Furthermore, some believed that systematic short breaks could be beneficial across all study sessions and found that frequent breaks were incompatible with their study habits. On the other hand, half of the participants felt that their self-regulated breaks were generally useful for all their studies; ‘*yes, I think it is easy to apply on all studies*’ (P36). However, some noted that their (self-regulated) break effectiveness depended on the specific study context and subjects, remarking, for instance, ‘*In some subjects maybe [self-regulated break works], but definitely not in all*’ (P28). Finally, regarding additional comments, some students in both conditions also noted that the study material (reading text) was either too challenging or not sufficiently engaging, with comments; ‘*The text was also very long, I felt like I was not going to finish it in the given time and that made me feel rushed and a bit uneasy*’ (P34), ‘*I found the text quite boring and difficult and that made it hard to concentrate*’ (P19).

## 4. Discussion

This study examined the efficacy of two different break types, the systematic short and self-regulated breaks, on university students’ learning outcomes, moods, and learning experiences during their online self-study environments. Regarding students’ learning outcomes (RQ1-H1a), the analysis did not reveal a statistically significant advantage for the systematic short-break condition in reading-comprehension scores. Bayesian analyses provided anecdotal to moderate evidence in favor of the null hypothesis (*BF*_01_ = 2.49), while the effect size was small (Cohen’s *d* = 0.24). However, this null finding should be interpreted with caution. The internal consistency of the reading-comprehension test was relatively low (*α* = 0.54), which may have reduced the sensitivity of the measure and attenuated potential differences between conditions. This finding contributes to a growing body of evidence suggesting that the types of break—systematic versus self-regulated—do not translate into performance differences in learning tasks (Biwer et al., 2023; Kao & Botelho, 2024). From a cognitive load and effort-regulation perspective, these findings suggest that while breaks may help prevent sustained overload, different break types may be equally effective for performance outcomes. Similarly, Biwer et al. (2023) and Smits et al. (2025) reported no significant performance differences between systematic and self-regulated breaks, even when learners selected their own study tasks. Despite differences in task type across Biwer et al. (2023), Smits et al. (2025), and the present study, the findings consistently suggest that no specific break strategy provides a clear advantage for objective learning performance. Instead, the effects of break type may be more evident in learners’ subjective experiences, shaping how they engage with and perceive their tasks. Moreover, the impact of different break types may depend not only on the break structure itself but also on the nature of the task (Lee et al., 2020). This highlights the need for further research into factors such as task complexity.

With respect to the students’ moods (RQ1-H1b), the results revealed that contrary to our initial hypothesis, self-regulated breaks were associated with more positive mood states than systematic short breaks in terms of energy, concentration, motivation, and distraction. Accordingly, with small to moderate effect sizes (*d* = 0.36–0.55; Cohen, 1988), participants in the self-regulated break condition reported feeling more energized, concentrated, and motivated, while also experiencing lower levels of distraction than those in the systematic short-break condition. However, these advantages should be interpreted cautiously, as the observed effects were generally small, limited to subjective mood-related outcomes, and did not translate into statistically significant differences in objective learning performance. Therefore, the benefits of self-regulated breaks appear to be primarily related to students’ subjective learning experiences rather than the improvements in learning outcomes. These findings align with Smits et al. (2025), who found that self-regulated breaks were associated with more favorable motivational experiences compared to structured breaks. However, they contrast with Biwer et al. (2023), who reported that self-regulated breaks led to higher distraction and lower motivation compared to systematic breaks. A plausible explanation for this divergence in findings may relate to differences in perceived flexibility and autonomy across study contexts. In our study, participants were required to follow a predetermined reading task with externally enforced break cycles. Unlike the self-regulated condition, students in the systematic short-break condition could not adjust the timing, frequency, or duration of their breaks to match their individual needs or study preferences. This difference in flexibility may have contributed to the more positive mood states reported in the self-regulated break condition. This interpretation is broadly consistent with self-determination theory (SDT), which proposes that autonomy-supportive environments tend to promote intrinsic motivation, vitality, and positive affect, whereas more controlling environments may undermine these outcomes (Ryan & Deci, 2017; Johansen et al., 2024). It is also in line with research on short breaks showing that greater autonomy in deciding when to take breaks can facilitate engagement and recovery (Kim et al., 2022). However, because perceived autonomy was not directly assessed, this explanation should be regarded as a plausible interpretation rather than a demonstrated mechanism. The qualitative findings further support this autonomy-based interpretation while also highlighting variation in students’ break preferences. Many students valued being able to decide when and how long to pause, suggesting that the self-regulated break condition may have been experienced as more autonomy-supportive. At the same time, some participants preferred shorter, more structured breaks, indicating that students may differ in the extent to which they value autonomy over externally structured break schedules. Such individual differences are consistent with perspectives on self-regulated learning, which view effort regulation as a dynamic process that depends on learners’ needs, preferences, and responses to task demands (de Bruin et al., 2020; Grund et al., 2024; Lohbeck & Moschner, 2022).

Beyond these effects, no significant differences were observed for certain mood states, including fatigue, boredom, stress, and interest, across the two break conditions. To explain the variation in mood states across different break strategies, Biwer et al. (2023) suggested that such effects may depend on the nature and specificity of the learning tasks. Similarly, Bennett et al. (2020) did not observe significant group differences in fatigue, vigor, or attention on break-taking. Research also emphasized that recovery needs fluctuate with task difficulty and individual factors, such as perceived demands and how individuals spend their break time; therefore, effective break strategies might be tailored to the specific task and learning context (Sonnentag & Zijlstra, 2006; Albulescu et al., 2022).

Regarding students’ experiences with study and break-taking behaviors, interestingly, despite the differences in the frequency and duration of breaks between the two break conditions, the average study times were remarkably similar. This suggests that variations in break structure did not significantly affect the total time spent studying, which is consistent with previous research (Biwer et al., 2023). Additionally, with respect to break use during study sessions, students in the systematic condition appeared to prefer taking fewer but longer breaks, rather than the shorter and more frequent breaks. Moreover, the results also showed that students engaged in diverse break activities, most commonly social media, followed by drinking, resting or chatting, and attending to physiological needs, indicating that breaks serve not only digital engagement but also physical, social, and restorative functions (Biwer et al., 2023; Kim et al., 2017). These findings underscore the role of breaks in addressing immediate personal needs and preferences, while also providing opportunities for mental or social engagement.

Concerning students’ experiences (RQ2), quantitative findings revealed higher acceptance, attractiveness, and preference scores for self-regulated breaks compared to systematic short breaks, while both break strategies showed similar levels of implementability and usability. This suggests that the flexibility in self-regulated breaks likely supports student preferences in certain tasks and contexts. These findings are consistent with the perspective that the benefits of breaks can vary significantly across individuals (Kim et al., 2022; A. Watson et al., 2019). Moreover, qualitative results on students’ experiences further showed that participants in both conditions preferred longer, but less frequent breaks during self-study. In the systematic short condition, frequent 3 min breaks were often criticized for disrupting study flow and concentration, whereas participants in the self-regulated condition favored breaks that fit better with their personal habits and time-management strategies. Importantly, these qualitative findings should not be interpreted as evidence against structured breaks in general. Rather, they may reflect dissatisfaction with the specific 12–3 schedule used in this study, particularly the short duration and frequency of the breaks. Several participants indicated that 3 min breaks were too short for meaningful recovery activities and interrupted concentration instead of restoring it. Although the average length of self-regulated breaks was only slightly longer (3 min 42 s) than the systematic short breaks (3 min), many participants perceived self-regulated breaks as more natural and less disruptive. These findings suggest that the effectiveness of break strategies may depend not only on break duration and study structure, but also on the degree of flexibility they provide. Allowing students greater flexibility to adapt the timing and duration of breaks to their individual needs and study routines may help support study flow, positive mood states, and overall learning experiences, regardless of whether breaks are self-regulated or externally structured (Bennett et al., 2020; Biwer et al., 2023).

Regarding the advantages and disadvantages of self-regulated and systematic short breaks, the results revealed both benefits and drawbacks, which varied between the two conditions. Specifically, despite criticisms of the frequent and rigid timing of systematic short breaks, these breaks were considered beneficial for maintaining motivation and focus, remarking, “*taking [systematic short] breaks more often did help me focus more, because I told myself I ‘only’ had to focus for 12 min before I could do something fun*” (P05). This perspective suggests that for certain learners, shorter break intervals may be attractive because they reduce the perceived opportunity costs of studying, as the next opportunity to engage in alternative, more rewarding activities feels closer and more attainable (Kurzban et al., 2013). In contrast, self-regulated breaks were widely regarded as advantageous in enhancing productivity and motivation due to their flexibility and alignment with individual preferences. Nevertheless, some participants noted that self-regulated short breaks could sometimes be insufficient for relaxation or distraction. These findings suggest that the type and length of breaks play a critical role in shaping students’ moods by mitigating negative emotions while enhancing positive affect (Albulescu et al., 2022; Bennett et al., 2020; Biwer et al., 2023; Kim et al., 2017, 2022).

Finally, regarding the applicability of the breaks, some participants recognized the conditional and contextual usefulness of systematic short breaks, whereas others found them incompatible with their existing study habits. In contrast, many participants considered self-regulated breaks to be more broadly applicable, although some emphasized that their effectiveness depended on the context and task. These findings align with the perspective that the effectiveness of breaks depends not only on their length or structure but also on their applicability to the specific context and conditions under which they are taken (Albulescu et al., 2022; Owen & Watts, 2026). Furthermore, the results highlight the importance of task types and individual differences in determining the applicability and preferences for break strategies.

In our preregistration, we planned to measure mental effort. However, the inclusion of this measure was unfortunately omitted during data collection. We acknowledge this deviation and report it transparently to ensure clarity and adherence to research integrity. Although self-regulated breaks were associated with more positive mood states, the absence of a mental effort measure prevents us from determining whether these differences were accompanied by differences in effort regulation.

## 5. Implications and Limitations

The present findings offer several practical implications for students and educators. First, self-regulated breaks may be particularly useful during tasks that require sustained concentration and motivation. A key advantage of self-regulated breaks is their flexibility, allowing students to adjust the timing and duration of breaks to their individual needs and study progress. A key advantage of self-regulated breaks is their flexibility, allowing students to adjust the timing and duration of breaks to their individual needs and study progress. Such flexibility may also support learners’ sense of autonomy during self-study, which may contribute to more positive learning experiences. However, because self-regulated breaks did not lead to superior learning performance, they should not be viewed as universally superior to structured break strategies. Second, systematic break schedules may still benefit students who prefer external guidance, have difficulty deciding when to take breaks, or need support in managing their study time. However, the effectiveness of systematic breaks may depend on the specific schedule used. The 12–3 schedule examined in this study was mostly perceived as too frequent and too short, suggesting that longer systematic formats may be worth exploring. Third, the findings suggest that no single break strategy is optimal for all learners and situations. Instead, break-taking strategies might need to be tailored to task characteristics, study duration, and individual learner preferences with flexibility in supporting students’ learning experiences.

The study also has several limitations. A key limitation of the present study concerns the duration and frequency of the systematic short breaks, half the length of the typical systematic break length (“Pomodoro” technique (Cirillo, 2018)). Many students in this condition primarily criticized the specific 12–3 schedule for disrupting their study flow and concentration, and expressed a preference for longer breaks. However, they did not criticize the concept of structured breaks itself. Future research could investigate whether longer structured breaks, such as the more common 25–5 schedule, or other systematic break formats better support cognitive recovery, learning experiences, and learning performance. Second, although the preregistration included plans to measure mental effort, this aspect was unfortunately overlooked in the study. As mental effort plays a crucial role in effort regulation (Biwer et al., 2023), the absence of this measure limits our ability to determine whether two break conditions differed in effort regulation during learning. Although self-regulated breaks were associated with more positive mood states, the mechanisms underlying these effects remain unclear. Future research could include mental effort to better understand how break-taking strategies influence effort regulation and learning. Moreover, although the effect size (*d* = 0.24) indicated a small advantage for the systematic short condition in reading comprehension scores, this difference was not statistically significant. Additionally, the relatively low internal consistency of the reading comprehension measure (Cronbach’s *α* = 0.54) may also have reduced the test’s sensitivity to detect potential differences between conditions. Future research employing more reliable assessment instruments and larger and more diverse samples could further examine the potential benefits of systematic break structures for students’ learning outcomes. Another limitation concerns the analysis of mood states. Each mood dimension was assessed using a single-item indicator after each break throughout the study session. Because participants in the self-regulated condition varied substantially in the number of breaks, with most participants taking only one break (see Table 1), they also contributed different numbers of mood assessments. Consequently, repeated-measures or multilevel analyses of mood trajectories were not feasible. Therefore, mood ratings were averaged across measurement occasions to compare participants’ overall mood experiences between conditions. Although this approach was appropriate for the study design, it may have obscured changes in mood over time. Future research could ensure comparable repeated mood assessments across participants to enable repeated-measures or multilevel analyses. The final limitation pertains to the learning task and duration of the study session. All participants completed a predetermined, challenging reading task (Flesch-Kincaid grade level 16.7) under systematic and self-regulated break conditions. In addition, the study session was limited to 60 min, which may not have been sufficient for fatigue or self-regulatory demands to accumulate and influence learning outcomes. As previous research suggests, the effectiveness of break-taking strategies may depend on task characteristics and study context (Albulescu et al., 2022; Biwer et al., 2023; Smits et al., 2025). Future research could therefore investigate break-taking strategies across tasks that vary in difficulty and duration, including longer study sessions, to better understand when different break formats are most beneficial.

## 6. Conclusions

This study explored the efficacy of systematic short and self-regulated breaks on university students’ moods, learning outcomes, and break-taking experiences during self-study. Although no statistically significant differences in task performance were observed between the two break conditions, this finding should be interpreted cautiously given the relatively low reliability of the comprehension measure. Self-regulated breaks nevertheless led to more positive subjective mood experiences, with students reporting higher motivation and concentration, and lower distraction. Moreover, although the 3 min systematic short breaks were often criticized for disrupting concentration and reducing flexibility, the qualitative findings suggested that this dissatisfaction was often directed at the specific 12–3 schedule rather than at the concept of structured breaks itself. In contrast, self-regulated breaks were generally preferred for their flexibility and alignment with personal study habits. Furthermore, students’ experiences emphasized the importance of flexibility, task and individual differences in shaping break effectiveness. In line with this, our results highlight the need to tailor break strategies to specific tasks to maximize their effectiveness.

## Figures and Tables

**Table 1:** Descriptive Statistics for Study Time, Number of Breaks, Break Duration, and Reading Comprehension Scores by Condition.

Questions	Systematic Short Breaks (n = 90) Mean (*SD*)	Self-Regulated Breaks (n = 86) Mean (*SD*)
Study time (in minute)	37.07 (12.92)	37.52 (12.59)
Number of breaks	3.08 (1.08)	1.79 (0.93)
Average break length (in minute)	3 (0.000) *	3.42 (2.60)
Reading comprehension test	12.93 (2.75)	12.23 (3.05)

Note. All reported *p* values are one-tailed. * Duration of systematic break was the same for all participants in the systematic short break condition.

**Table 2:** Students’ Moods.

Moods	Systematic Short Breaks (n = 90) Mean (*SD*)	Self-Regulated Breaks (n = 86) Mean (*SD*)
Interest	2.18 (0.73)	2.33 (0.85)
Energy	2.50 (0.75)	2.77 (0.76)
Concentration	2.46 (0.69)	2.78 (0.79)
Motivation to study	2.21 (0.77)	2.68 (0.94)
Fatigue	2.50 (0.94)	2.46 (0.87)
Bored	2.77 (0.94)	2.56 (0.96)
Distraction	2.69 (0.81)	2.24 (0.83)
Stress	1.54 (0.80)	1.59 (0.87)

Note. All reported *p* values are one-tailed and were adjusted using the FDR correction procedure.

**Table 3:** Acceptability and Feasibility of the Breaks.

Statements	Systematic Short Breaks (n = 90) Mean (*SD*)	Self-Regulated Breaks (n = 86) Mean (*SD*)
Acceptance	3.15 (0.95)	3.54 (0.90)
Attractiveness	2.80 (1.05)	3.36 (0.93)
Preference	2.73 (1.10)	3.41 (1.03)
Implementability	3.42 (0.92)	3.66 (0.94)
Usability	3.66 (1.00)	3.86 (0.84)

Note. All reported *p* values are one-tailed and were adjusted using the FDR correction procedure.

## Data Availability

The data that support the findings of this study are available on request from the corresponding author. The data are not publicly available due to privacy or ethical restrictions.

## Appendix A. Sample Reading Comprehension Test Items

Choose the best answer for each question based on the text “Ground water and climate change” you read.

Item 1. What is the primary source of freshwater withdrawals globally?

(a) Surface water
(b) Groundwater (Correct answer)
(c) Desalinated water
(d) Rainwater harvesting

Item 2. Which term refers to groundwater that was recharged during cooler, wetter climates thousands of years ago?

(a) Ancient groundwater
(b) Fossil groundwater (Correct answer)
(c) Prehistoric groundwater
(d) Archaic groundwater

Item 3. What factor strongly influences recharge in semi-arid environments?

(a) Snowmelt
(b) Evapotranspiration
(c) Heavy rainfall (Correct answer)
(d) Groundwater depletion

Item 4. What is the primary source of uncertainty in projecting the impacts of climate change on groundwater recharge?

(a) Land use change
(b) Human population growth
(c) Greenhouse gas emissions
(d) Climate model projections (Correct answer)

Item 5. What is the main challenge in evaluating groundwater responses to climate variability and change?

(a) Lack of groundwater observations (Correct answer)
(b) Lack of satellite data
(c) Lack of funding for research
(d) Lack of technological advancements

Item 6. How does climate variability affect groundwater recharge?

(a) It has no effect on recharge rates
(b) It decreases recharge rates (Correct answer)
(c) It increases recharge rates
(d) It stabilizes recharge rates

Item 7. What are proposed adaptation strategies for sustaining groundwater resources in the face of climate change?

(a) Managed aquifer recharge and conjunctive use of surface water (Correct answer)
(b) Increasing groundwater abstraction and deforestation
(c) Desalination and reducing groundwater monitoring efforts
(d) Drilling deeper wells and relying on natural groundwater replenishment

## Appendix B. Themes, Codes, and Example Quotes

**Theme: Differences and Similarities in Break-Taking**	
**Codes**	**Example Quotes**
Preference for longer, but fewer breaks	“I usually study for longer periods 40 min to 1 h and then take a 20 min break” (P24, Systematic short break condition) “During my usual studying I tend to take very little breaks and work for long periods at once” (P65, Self-regulated condition)
Short and frequent breaks	“The breaks came relatively quickly, which meant that I needed time again to get focused on the text I was reading, while only having limited time after I got my concentration back on the text again” (P17, Systematic short break condition)
No difference for break-taking	“It was not different. I always implement regular breaks as I know this improves my focus!” (P45, Self-regulated condition)
**Theme: Break influences on study efficiency and focus**	
**Codes**	**Example Quotes**
Negative influence on study	“I think it interrupted me too much. Fewer but longer breaks suit me better.” (P27, Systematic short break condition)
Increased focus	“It forced me to focus for shorter periods of time and to re-focus after each study break.” (P18, Systematic short break condition) “It helped to give strengths and came back more focused to continue the reading” (P69, Self-regulated condition)
Increased concentration	“It allowed me to regain concentration to read again. The text was quite long, so I needed the break to stay focused” (P81, Self-regulated condition)
Decreased concentration	“I found it [systematic short break] too short, my concentrations were broken each time” (P08, Systematic short break condition)
Positive influence on study	“It [self-regulated break] optimized my time span for the reading” (P26, Self-regulated condition)
**Theme: Perceived advantages of break-taking**	
**Codes**	**Example Quotes**
Increased focus, concentration	“I felt very concentrated and rushed when starting a study session. This was a positive influence” (P33, Systematic short break condition)
Increased motivation and productivity	“Yes, I feel like the breaks give me some motivation to keep going” (P44, Self-regulated condition) “It refreshed my mind and allowed me to get back into reading the text. I felt like the text was really overwhelming and not appealing, so I found myself zoning out. The break also allowed the information to really process in my brain” (P20, Systematic short break condition)
No significant advantage	“Personally, this method [systematic short breaks] of taking breaks has no advantage for me. My usual break-taking is far more fruitful as it allows me to ‘cleanse’ my mind from the previous topic and prepare it for the following one…” (P35, Systematic short break condition)
Positive affect	“Going outside or getting something to drink or eat usually has a positive impact” (P23, Self-regulated condition)
**Theme: Perceived disadvantages of break-taking**	
**Codes**	**Example Quotes**
Disrupt the focus	“The breaks made me stress more, and they disrupted my focus quite a bit” (P51, Systematic short break condition)
Disrupt the study flow	“This 3 min break-taking method has, in my opinion many disadvantages. Firstly, the time to study is very short, so when one actually starts understanding what is going on in the reading material, the time is usually up and the break deflates all the interest/motivation to go on.” (P34, Systematic short break condition)
No disadvantage	“I have not, this was very effective for me” (P45, Self-regulated condition)
Too short break	“The break was too short and did not allow for room for anything more relaxing” (P19, Systematic short break condition)
Decrease concentration	“I noticed that after a break my concentration was less” (P79, Self-regulated condition)
**Theme: Break applicability to study routine**	
**Codes**	**Example Quotes**
Applicable across conditions	“Not [ applicable] all. For instance, for exams I like to study longer periods of time and then take a big break, however for daily assignments etc…” (P31, Self-regulated condition) “I think it depends. For readings articles and books, it [systematic short break] is a nice way to recharge as you can get tired from reading for long sessions but for other assignments such as writing, it is nice to just let the ‘study time’ flow on until I feel that I need a break, rather than having pre-planned breaks” (P22, Systematic short break condition)
Applicable for all studies	“Yes I think it [self-regulated break] is easy to apply on all studies” (P39, Self-regulated condition)
Not applicable for all studies	“No, this method is not applicable to my studies at all. I am used to studying uninterrupted for longer periods of time, allowing me to really focus on what I am studying and get into the subject properly. Here, I was forced to stop focusing on the text always quite abruptly…” (P51, Systematic short break condition)
